# Blood-based molecular biomarkers for Alzheimer’s disease

**DOI:** 10.1186/s13041-019-0448-1

**Published:** 2019-03-28

**Authors:** Henrik Zetterberg, Samantha C. Burnham

**Affiliations:** 1 Department of Psychiatry and Neurochemistry, Institute of Neuroscience and Physiology, he Sahlgrenska Academy at the University of Gothenburg, Mölndal, Sweden; 2 Clinical Neurochemistry Laboratory, Sahlgrenska, University Hospital, Mölndal, Sweden; 3 Department of Neurodegenerative Disease, UCL Queen Square Institute of Neurology, Queen Square, London, UK; 4 UK Dementia Research Institute at UCL, London, UK; 5 CSIRO Health and Biosecurity, Parkville, Victoria 3052 Australia; 6 Centre of Excellence for Alzheimer’s Disease Research and Care, School of Medical and Health Sciences, Edith Cowan University, Joondalup, Western Australia 6027 Australia

**Keywords:** Alzheimer’s disease, Serum, Plasma, Blood, Biomarkers, Amyloid, Tau

## Abstract

A major barrier to the effective conduct of clinical trials of new drug candidates against Alzheimer’s disease (AD) and to identifying patients for receiving future disease-modifying treatments is the limited capacity of the current health system to find and diagnose patients with early AD pathology. This may be related in part to the limited capacity of the current health systems to select those people likely to have AD pathology in order to confirm the diagnosis with available cerebrospinal fluid and imaging biomarkers at memory clinics. In the current narrative review, we summarize the literature on candidate blood tests for AD that could be implemented in primary care settings and used for the effective identification of individuals at increased risk of AD pathology, who could be referred for potential inclusion in clinical trials or future approved treatments following additional testing. We give an updated account of blood-based candidate biomarkers and biomarker panels for AD-related brain changes. Our analysis centres on biomarker candidates that have been replicated in more than one study and discusses the need of further studies to achieve the goal of a primary care-based screening algorithm for AD.

## Introduction

Alzheimer’s disease (AD) is characterized by the accumulation of extracellular amyloid β (Aβ) plaques, intraneuronal inclusions (neurofibrillary tangles) composed of truncated and phosphorylated forms of the microtubule-stabilizing protein tau, dystrophic neurites, loss of synapses and neurons, and a prominent gliosis that involves changes in the morphology and function of microglia and astrocytes [1]. Currently, we have validated biomarkers for amyloid pathology (Aβ positron emission tomography [PET] and the ratio of 42 over 40 amino acid long Aβ cerebrospinal fluid [CSF Aβ42/Aβ40]) [2], as well as tau dysfunction and aggregation (cerebrospinal fluid [CSF] total or phosphorylated tau and tau PET) [3]. CSF and PET examinations are, however, far from standard tests in general practice and, given the high prevalence of the disease, alternatives such as blood-based biomarkers would represent a significant development, even as screening tools to determine who should be referred to a memory clinic for specific testing. Here, we review recent developments in the field of blood biomarkers for AD and discuss the results in the context of the quests to improve diagnostic algorithms and facilitate clinical trials of novel candidate drugs.

### Blood biomarkers – general considerations

Measuring biomarkers for brain diseases in the blood poses a number of challenges that demand sensitive and specific assays and careful validation work. Brain-derived biomarkers are typically present at relatively low concentrations in the blood because of the blood-brain barrier preventing free passage of molecules between the CNS and blood compartments. In addition, some of the biomarkers related to AD pathology are expressed in non-cerebral tissues, which may confound their measurement in the blood. Further, in blood, there may be heterophilic antibodies (endogenous antibodies that react with the antibodies of the immunochemical test to measure the biomarker), which may give falsely high or low results. These types of antibodies are much less of a problem in CSF where antibody levels are much lower. Finally, the analyte of interest may undergo proteolytic degradation by various proteases in plasma. Below, we discuss recent developments of biomarkers for the pathological processes involved in AD. We discuss biomarker tests that are directed against a single pathological change and biomarker panels that may reflect tissue reactions to such changes.

### Targeted blood-based biomarkers

#### Plasma Aβ

Early results on plasma Aβ, described in the AlzBiomarker database (https://www.alzforum.org/alzbiomarker), revealed no consistent change in either plasma Aβ42 or Aβ40 in AD [4]. This result was most likely due to assay-related difficulties; plasma Aβ measurements may be influenced by matrix effects (mainly other plasma proteins binding Aβ) and the analytical sensitivities of the first assays did not allow for diluting away such matrix effects. In 2011, an ultrasensitive Single molecule array (Simoa) assay for Aβ42 was published [5]. A correlation of plasma with CSF Aβ42 emerged and the improved analytical sensitivity clarified that the ratio of Aβ42 to Aβ40 in plasma was reduced in amyloid PET-positive individuals in a manner similar to CSF Aβ42/Aβ40, although with less marked separation [6, 7]. A few years ago, reliable immunoprecipitation mass spectrometry (IP-MS)-based assays for Aβ40 and Aβ42 were described showing a decrease in the plasma Aβ42/Aβ40 ratio (similar to the CSF test) with around 90% diagnostic accuracy [8, 9]. These are highly promising developments that have spurred several method comparison and standardization studies that are just about to start. These new assay formats do not solve the confounding problem with non-cerebral expression of Aβ, e.g., in blood platelets [10], which affects the specificity of the test for cerebral Aβ plaques, but still represent an important step forward.

#### Plasma tau

In the dementia stage of AD, plasma tau concentrations, measured using ultrasensitive assays, are increased compared with cognitively normal control individuals, but not as clearly as in CSF [11], which is a well-replicated finding [4]. There is little clarity on the application of these findings to intermediary participants in the mild cognitive impairment (MCI) stage of the disease with reports being less clear [12]. Nevertheless, in a recent paper, Mielke and colleagues examined the relationship of plasma T-tau concentration, determined by Simoa, with cognitive decline in 458 participants from the Mayo Clinic Study on Aging and found high plasma T-tau associated with faster clinical disease progression [13]. Another study prospective cohort study used data from the US community-based Framingham Heart Study with replication in the Memento study, and found that plasma T-tau was strongly associated with incident AD dementia [14]. Mielke et al. also developed an assay for tau phosphorylated at amino acid 181 [15]. Plasma P-tau concentration, measured using this assay, was associated with both Aβ and tau PET, as well as with other AD-associated phenotypes [15]; these associations were stronger than those obtained using the plasma T-tau test. The general findings of this study resonate well with two studies showing increased plasma P-tau concentration in AD, as determined using immunomagnetic reduction technology [16] and Simoa [17], respectively.

#### Plasma neurofilament light

A well-replicated biomarker for neurodegeneration in AD is plasma neurofilament light (NfL), which is an intra-axonal structural protein that leaks into body fluids upon axonal injury irrespective of cause [18]. Serum or plasma levels of NfL (either matrix has good efficacy) correlate strongly with CSF NfL, are increased in several non-AD neurodegenerative diseases and are increased in both familial and sporadic AD [18]. In familial AD, the levels increase in the time window around 10 years prior to expected clinical disease onset [19]; in sporadic disease the changes may appear slightly later and may also be influenced by age-related changes (NfL concentration increase around 3% per year in CSF in normal aging [20]), or non-AD pathologies [21]. Altogether, plasma or serum NfL may be a reliable blood biomarker for neurodegeneration in AD and other neurodegenerative diseases.

### Panels of blood-based biomarkers

#### Protein biomarker panels

It has been suggested that panels of markers may outperform single candidate markers for diagnosing, prognosing and characterising AD [22]. Thus, a number of multivariate blood-based biomarker panels have been proposed for classifying the disease and its pathology. A large number of reports show efficacy of stratifying cases from controls using multivariate panels of protein markers. Ray et al. published one of the first of these studies, which identified 18 plasma proteins whose combined signature could distinguish AD patients from controls with 90% accuracy, as well as identify MCI patients who were at risk for progression to AD within five years [23]. The same group subsequently published results from independent samples detailing that many of the 18 proteins originally identified were also associated with CSF levels of Aβ and tau [24]. However, other attempts to replicate the original findings have not been successful. Marksteiner and colleagues found that only five out of 16 of the original 18 markers had significantly different concentrations in MCI and AD patients vs. controls [25]. They found the sensitivity and specificity of the panel for identifying AD and MCI to be 65–75% and 52–63%, respectively [25]. Similarly, Soares et al. found the diagnostic accuracy of the panel to be 60% [26], whilst Björkqvist et al. reported an area under the curve (AUC) of 63% [27]. Soares et al., after failing to replicate efficacy in the Ray panel, proposed an alternative 89-analyte panel with a diagnostic accuracy of 70% [26]. Subsequently, there have been numerous contributions of multivariate signatures for differentiating AD and/or MCI patients from controls [28–39], as well as a number of review articles [22, 40–43]. Two studies of most note include contributions by O’Bryant and colleagues [44] and Doecke and colleagues [45] who identified algorithmic signatures of multivariate analytes in large, well-characterised cohorts with AUC characteristics of at least 93% in serum and plasma, respectively. Doecke et al. also validated their signature in a second cohort with an AUC of 85% [45]. Many of the analytes identified across these studies are related to amyloidosis and/or inflammation, but still there has been a notable lack of harmonisation and replication of the findings. This is an area of research that has been much scrutinised, with a number of considerations and recommendations being made by O’Bryant and colleagues regarding study design, pre-analytical protocols and assay-related issues [46–49].

#### Panels of blood-based biomarker-associated disease phenotypes

Further to case-control studies, blood-based biomarker panels have been designed to estimate disease-related phenotypes, such as cognitive decline, brain atrophy and neocortical Aβ deposition. Inflammatory markers [50–52], as well as proteins associated with the complement cascade [53], have reported associations with cognitive performance, cognitive decline and clinical progression. Most of these contributions also examined the association of proteomic signatures with brain volumetry finding similar signatures [50, 54–56]. Reports suggest that such signatures are able to explain approximately one third of the between subject variation observed in brain volumes [54, 57].

Blood biomarkers able to identify AD in its earliest stages are predicted to have the most impact for use as a screening tool. The early timing of abnormal levels of neocortical Aβ deposition make it the ‘gold standard’ for early identification of disease, and therefore blood signatures associated with neocortical Aβ deposition have received considerable interest. Burnham et al. identified six plasma proteins that contributed to a signature that was able to estimate levels of neocortical Aβ deposition in the AIBL cohort with a sensitivity and specificity of 80 and 82%, respectively [58]. Validation of this signature in ADNI samples resulted in 79% sensitivity and 76% specificity [58]. The same group published follow-up results 54 months later, detailing the predictive ability of the same signature for identifying progression towards disease; cognitively normal individuals considered at risk by the signature progressed to MCI or AD with an odds ratio of 2.4 in comparison to those considered not at risk [59]. MCI participants considered at risk by the signature progressed to AD with an odds ratio of 12.3 in comparison to those considered not at risk [59]. Kiddle et al. similarly reported a signature that was associated with neocortical Aβ deposition, identifying 13 markers which were able to explain over 30 % of the between subject variation observed in neocortical Aβ deposition [60]. Other studies with the same aim of inferring neocortical Aβ deposition from a blood biomarker signature have reported efficacies of between 58 and 78% [61–63]. A replication study by Voyle and colleagues found pancreatic polypeptide and IgM to correlate with neocortical Aβ deposition, with IgM also correlating with neocortical Aβ deposition within cognitively normal participants [63]. Again, the signatures across these contributions aligned with both inflammatory response and the complement cascade.

#### Metabolomics

Recent advances in nuclear magnetic resonance and mass spectroscopy, coupled with high performance liquid/gas chromatography have allowed the dynamic evaluation of thousands of metabolites, reflecting functional networks of downstream changes of the genome, transcriptome and proteome. Metabolic changes in plasma demonstrating a separation between controls and AD were reported by Greenberg et al. [64], with the two most influential metabolites being glycerophosphocholine and d-glucosaminide. Another contribution from Oresic et al. demonstrated metabolites that were differentially expressed in MCI participants who did and did not progress to AD within a 2-year time frame, with this finding being specifically driven by 2,4-dihydroxybutanoic acid [65]. Trushina and Mielke discuss results suggesting beta-alanine, aspartate and aspargine, alanine, l-cysteine, l-methionine, methionine–cysteine–glutamate, l-arginine, lysine and bile acid biosynthesis and metabolism were significantly different between controls and AD [66].

With links between cardiovascular disease and AD being drawn, as well as some promising results from lifestyle intervention on cognitive performance [67], lipid evaluation has become another area of interest in the search for blood-based biomarkers of AD. Two recent pilot studies have shown levels of lipids to differ significantly between mutation carriers and non-carriers in a study on familial AD [68], as well as AD and controls in a sporadic AD study [69]. However, neither study was able to use these profiles to accurately discriminate between the respective classes. Mapstone and colleagues were able to successfully discriminate AD from controls with 90% sensitivity and specificity in two separate studies. Firstly, using a panel of 10 lipids [70] and secondly with a panel of 22 lipids combined with an amino acid and a biogenic amine [71]. However, an independent group was unable to validate the findings from the 10-lipid panel in larger studies [72].

#### miRNA biomarker panels

Epigenetics are also of increasing interest to the field, with gene regulation by micro RNA (miRNA) representing one such focus for biomarker discovery. It is hypothesised that miRNAs are transported within liposomes, HDLs, exosomes and other proteins protecting the miRNA from degradation. Leidinger et al. first reported a panel of 12 miRNAs able to discriminate AD from controls with an accuracy of 93% [73]. The panel also had the ability to discriminate AD patients from patients with MCI, multiple sclerosis, parkinsonism, major depression, schizophrenia, and bipolar disorder with accuracies of 74–78% [73]. Other studies identifying panels of miRNAs have been reported to discriminate between AD patients and controls with accuracies between 75 and 95% [74–76]. A recent review of miRNAs as biomarkers for AD is provided by Nagaraj and colleagues [77] where they report 136 individual miRNAs to be significantly altered between AD and control states from the literature. Of these 136 miRNAs, 36 were independently validated and reported in two or more contributions, with hsa-miR-125b being reported the most, 6, times.

#### Exosomes

This is a hot and controversial topic in the field of blood-based biomarkers for AD. Initial reports, in which neuronally derived exosomes were isolated from serum using antibodies against CNS-enriched antigens, lysed and analysed for AD-related biomarkers, showed promising results [78, 79], but the protocols are hard to follow and it is at the moment difficult to draw any strong conclusions on where this field is going.

### Future perspective

As instruments for quantifying blood biomarkers become more sensitive and the understanding and implementation of standardisation procedures for sample processing and analysis are increased, the field moves ever closer to the quest for blood biomarkers in AD. These improvements appear to have provided a shift in the most recent literature, away from multiplexed assays that were popular approximately 5 years ago, back full circle to single candidate markers that were also the focus of investigations 10–15 years ago. The candidate markers showing the most promise as markers for AD are plasma Aβ42/Aβ40 ratio, tau and NfL but there is also promising data on new panels that may represent tissue responses to AD-related pathologies. However, replication and more detailed analyses are required to understand their merits as potential screening, diagnostic, prognostic or monitoring markers of the disease. An important goal will be to develop a screening/triage algorithm based on blood tests for Aβ pathology and neurodegeneration that could be implemented in primary care settings and used for the effective identification of individuals who could be referred for further testing using CSF and PET markers and potential inclusion in clinical trials or future approved treatments. To validate such candidate algorithms, the AD biomarker research community needs to collaborate with primary healthcare specialists to establish new primary care-based cohorts that are evaluated using currently available gold standard measures of AD pathology (CSF and/or amyloid PET in specialised memory clinics) following application of the index test (the blood-based biomarker algorithm). When validated, such a diagnostic algorithm would facilitate AD drug development and prepare effective clinical pathways for the advent of disease-modifying therapies targeted against amyloid pathology.

## Abbreviations

AD: Alzheimer’s disease
AUC: Area under the curve
Aβ: Amyloid β
CSF: Cerebrospinal fluid
IP-MS: Immunoprecipitation mass spectrometry
MCI: Mild cognitive impairment
miRNA: Micro RNA
NfL: Neurofilament light
PET: Positron emission tomography
Simoa: Single molecule array
